# Cancer and Aging - the Inflammatory Connection

**DOI:** 10.14336/AD.2016.1230

**Published:** 2017-10-01

**Authors:** Adar Zinger, William C Cho, Arie Ben-Yehuda

**Affiliations:** ^1^Department of Medicine, Hadassah-Hebrew University Medical Center, Jerusalem, Israel; ^2^Department of Clinical Oncology, Queen Elizabeth Hospital, Kowloon, Hong Kong

**Keywords:** aging, cancer, senescence, inflammation, immunosenescence, autophagy

## Abstract

Aging and cancer are highly correlated biological phenomena. Various cellular processes such as DNA damage responses and cellular senescence that serve as tumor suppressing mechanisms throughout life result in degenerative changes and contribute to the aging phenotype. In turn, aging is considered a pro-tumorigenic state, and constitutes the single most important risk factor for cancer development. However, the causative relations between aging and cancer is not straight forward, as these processes carry contradictory hallmarks; While aging is characterized by tissue degeneration and organ loss of function, cancer is a state of sustained cellular proliferation and gain of new functions. Here, we review the molecular and cellular pathways that stand in the base of aging related cancer. Specifically, we deal with the inflammatory perspective that link these two processes, and suggest possible molecular targets that may be exploited to modify their courses.

## 1. Introduction

Age is the single most significant risk factor for cancer development, with the majority of cancer cases being diagnosed after the age of 65 [1]. The most common cancer types are prostate cancer in men, breast cancer in women, followed by lung and colorectal cancer in both sexes. When comparing the probability to develop invasive cancer before the age of 50 and after the age of 70, a dramatic 3.5, 36, 28 and 12 fold increase is demonstrated in breast, prostate, lung and colorectal cancer respectively [2]. Aging is a biological process that occurs in virtually all organisms, and is characterize by a progressive organ loss of function and decline in tissue renewal capacity [3, 4]. This stands in striking contrast to the unlimited proliferation, resistance to apoptosis and gain of new, albeit aberrant, functions that are among the hallmarks of cancer [5, 6]. Advances in the research of both cancer and aging have started to decipher this inherent dichotomy, and shed some light on the common molecular and cellular pathways of these supposedly contradictory processes.

The antagonistic pleiotropy hypothesis states that genes that cause degeneration and aging survived the evolutionary selection because they confer beneficial effects earlier in life during reproductive years [7, 8]. Indeed, many of the biological processes that were linked to deleterious effects during aging bear crucial pro-survival functions during earlier stages of life. Cellular senescence is a striking example for this paradigm. Senescence was shown to be an essential process during embryonic development [9]. It is also a strong tumor suppressor mechanism that prevents damaged cells that harbor potentially oncogenic mutations from proliferating [10, 11]. That very same process was found to be an important causative factor in multiple age-related pathologies [12], including cancer [13]. And so, one of the challenges in aging and cancer research is the exploration of the changing conditions that turn a tumor- suppressor mechanism into a pro-tumorigenic one.

Overt inflammation is most commonly triggered by exogenous pathogens. However, inflammation may be triggered by multiple other stressors such as DNA damage, UV radiation and physical trauma. These stimuli can cause low grade sterile inflammation which involves not only immune cells, but also other types of cells such as epithelial cells and fibroblasts. We now know that those inflammatory processes accompany and modulate multiple biological processes, including cancer and aging-related pathologies [14-17].

Along with the deterioration of other tissues during the aging process, immune system function declines as well. Thymic involution has an important contribution to this decline. Consistent with the antagonistic pleiotropy principle, it is suggested that thymic atrophy provides beneficial effects early in life including reducing the chance of foreign pathogens to be recognized as “self” [18], reduce energy expenditure and minimize autoimmunity [19], while later in live it contributes to immunosenescence [20, 21]. Immune surveillance is a key factor in preventing cancer progression; therefore immunosenescence is another important factor that links tumorigenesis and aging [22].

**Figure 1.**
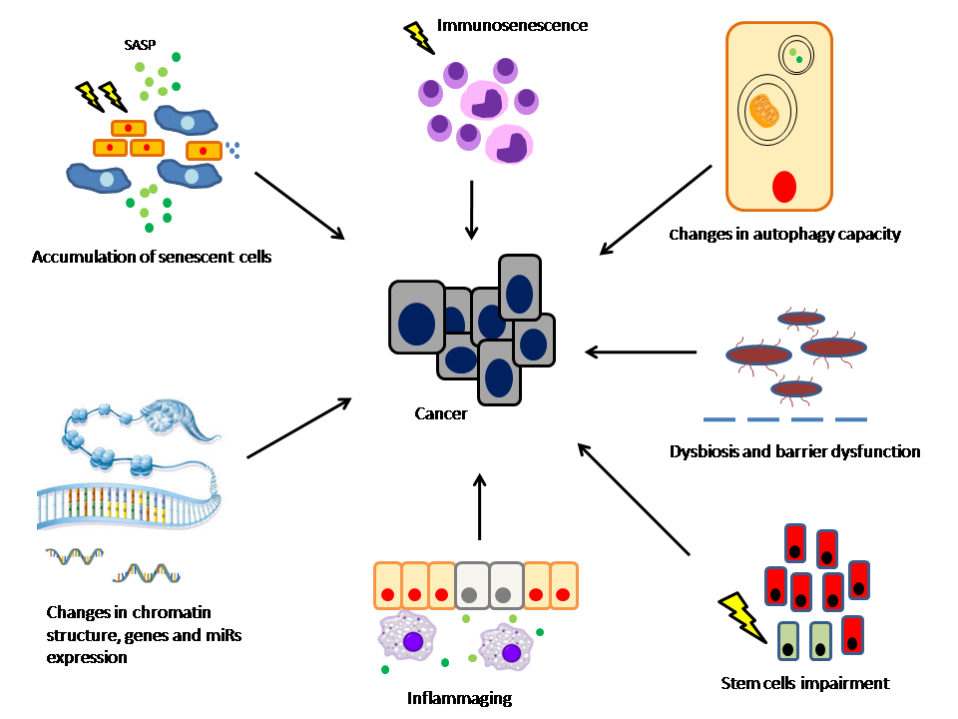
Aging-related cancer Exposure to various endogenous and exogenous stressors throughout life results in multiple cellular and tissue function changes. Accumulation of senescent cells in the tissue is associated with tissue degeneration and SASP-related changes of the microenvironment. Functional changes in aging immune system along with DAMPs-associated immune responses contribute to the ensemble of inflammatory processes that accompanies the aging process, so-called “inflammaging”. This unique inflammatory network joins intracellular processes including changes in chromatin function and reduction in autophagy capacity, and to changes in the microbiome and intestinal barrier dysfunction, to create a pro-tumorigenic environment.

Here, we review the different cell autonomous and non-cell autonomous mechanisms that link aging and cancer (Fig. 1). We will explore the inflammatory perspective, which provide plausible explanations for the common ground, as well as for some of the contradictories between these two biological processes.

## 2. DNA damage response- balance between cancer prevention and aging promotion

The key event that leads to cancer initiation and progression is DNA damage, which results from constant attacks by genotoxic agents throughout life. These insults might result in genome instability and mutation accumulation [23, 24]. The damaging agents may be exogenous, e.g. environmental exposure to UV light [25], ionizing radiation [26] or genotoxic chemicals [27]. They may also be endogenous factors including reactive oxygen species (ROS), which are byproduct of multiple metabolic cellular processes [28], as well as a result of flaws in the cellular DNA replication machinery [29] or telomere dysfunction [30]. There are various types of DNA damage, including-single strand breaks, double-strand breaks, intrastrand and interstrand crosslinking, which differ in their causative agents and in the cellular response they initiate to repair the damage [31].

As DNA damage is the initiating event in tumorigenesis, one of the first lines of defense against malignant transformation is the DNA damage response (DDR). DDR is a highly conserved cellular mechanism of cell cycle checkpoints. DNA damage results in activation of a cascade of kinases, including ATM, ATR CHK1 and CHK2, which ultimately results in stabilization of the tumor suppressor p53 [32, 33]. DDR can cause a temporary cell cycle arrest that allows damage repair, or in case of damage that cannot be resolved- cellular senescence or apoptosis.

p53 is a key player in the DDR and one of the most important cellular gatekeepers in the prevention of uncontrolled growth and division [34]. Its activation induces signalling in several downstream effector arms. p21 is one of the most important targets of p53. It is a potent cyclin-dependent kinase (CDK) inhibitor, and a major regulator of the G1/S checkpoint. Its activation results in cell cycle arrest, and in case of continuous damage eventually leads to cellular senescence [35]. Activation of another downstream arm which includes PUMA, BAX and BAK induces apoptosis [36]. Both senescence and apoptosis are strong tumor suppressors that withhold damaged cells from undergoing malignant transformation, and are important pro-survival mechanisms in the whole organism level. However, by their nature, they also lead to tissue degeneration and function loss, as well as depletion of stem cells and impaired renewal capacity- all of which are hallmarks of the aging process [37]. Indeed, studies using different mouse models for p53 hyperactivation demonstrate increased resistance to tumors, but at the same time- premature aging and reduced longevity. Experiments in transgenic mice overexpressing the short isoform of p53- p44, showed increased activity of WT p53 which resulted in reduced tumor incidence, accompanied by slower growth rate and accelerated aging [38]. Similarly, a p53^+/m^ mice that harbor p53 truncated mutant which drives higher p53 activity displayed stem cell dysfunction and pre-mature aging phenotype, along with lower tumor frequency [39]. Of note, in these studies p53 hyperactivation was achieved using short isoforms that were not subjected to the physiologic regulation of p53. However, a ’super p53’ model showed different results. In this model, full size p53-transgene was added to the endogenous alleles and retained normal regulation as the WT p53. Unlike the previous hyperactivation models, theses mice demonstrated relative resistance to tumors without premature aging [40]. These data may suggest that chronic dysregulated activation of p53, rather than increased pulsate activation in response to intermittent stress, leads to accelerated aging [41]. Since greater number of cells and cell divisions increases the chance for mutation resulting in malignant transformation, it could have been speculated that across species, mammals with higher body mass and longer life span would have increased rate of cancer. However, studies show that this correlation does not exist. A study that examined elephants, which in spite of their large body size and longevity have low rates of malignancy, found that they harbor multiple copies of the TP53 allele. Indeed, *in vitro* studies showed increased apoptosis of elephant peripheral blood lymphocytes in response to ionizing radiation compared to human cells. This lead to the speculation, that the low malignancy rate in elephants is a result of more efficient p53 activation in response to DNA damage [42].

Functional decline of adult stem cells is an important component of the aging phenotype. Molecular pathways that control self-renewal capacity, such as the Wnt signaling pathway, are often de-regulated in aged organisms. Aberrant Wnt signaling which characterizes many cancer types was also linked to reduce renewal capacity of normal stem cells during aging. Accumulation of DNA damage and activation of tumor suppressor signaling pathways is another important factor underlying the reduced ability of the stem cells to regenerate and repair damaged tissues. The change in the aging stem cells population does not necessarily manifests as quantitative reduction in their number, but rather as a qualitative change and reduced functional capacity. One of the phenomena observed in the aging stem cells population is clonal drift. In the hematopoietic system, for example, aging affects greatly on compartments. This change is attributed, at least partially, to decline in the frequency of the lymphoid lineage committed stem cells, and the increase in the myeloid lineage committed ones [43]. It has been proposed that inherent differences in the DDR serve as driving forces behind this drift in stem cell clones. These differences may be the result of increased or decreased DNA repair capacity in certain subpopulations, distinct checkpoint responsiveness, and variable up- regulation of ’eat me’ signals in response to accumulating damage [44].

Although DDR is essentially a cell autonomous process that serves as an internal quality assurance mechanism, its activation consequences go beyond the single cell boundaries. Induction of senescence and accumulation of danger signals that follow DNA damage and DNA damage responses contribute to multiple systemic processes in the whole organism level.

## 3. Aging, cellular senescence and cancer

Cellular senescence is defined as an irreversible arrest of cell proliferation. It was first described in 1961 by Hayflick and Moorhead who demonstrated that non-transformed tissue culture cells can only divide a limited number of times [45]. Further *in vitro* studies showed an inverse proportion between the maximal number of cell divisions, and the age of the cell’s donor [46]. This phenomenon, termed replicative senescence, is attributed to telomeres attrition which triggers continuous DNA damage response and cell cycle arrest [47]. Later studies showed that not only repeated replication, but also other stressors, such as ROS accumulation [48], persistent oncogene activation [49] and chromatin modifications [50] can enter the cell into a senescent state. These different stressors converge into activation of two main tumor suppressor signaling pathways: p53/p21 and p16^INK4a^/pRB, which results in growth arrest, and in case of persistent stimuli leads to senescence [6].

### 3.1. Senescence and aging: correlation or causation?

Senescence was first described *in vitro* in tissue culture cells, but the absence of specific markers hindered the study of its *in vivo* relevance. Later, several markers including senescence-associated β-galactosidase (SA-β-Gal) and p16^INK4a^ were identified as reliable biomarkers for senescence, enabling to examine it *in vivo* [51]. Senescent cells were shown to accumulate in aged tissues of rodent and primate models, as well as in human tissues. Their accumulation was linked to multiple age-related pathologies, including atherosclerosis, Alzheimer’s disease and osteoarthritis [46]. Demonstrating the temporal relations between these phenomena lay the foundation to the hypothesis that cellular senescence has a crucial role in the aging phenotype of the whole organism. However, *in vivo* studies were further hampered by the fact that depleting of the main senescence effectors p16^INK4a^ and p53 in rodent models lead to premature cancer-related death before reaching the point in which aging related pathologies were expected to develop [52]. A progress in proving the causative relation between senescence and aging was made in studies that used the progeroid mouse model BubR1^H/H^. BubR1 is a mitotic checkpoint protein involved in spindle checkpoint function and chromosomal segregation. Hypomorphic BubR1 mice show premature separation of sister chromatids which leads to progressive aneuploidy. They develop a characteristic progeroid phenotype with high p16^INK4a^ expression in skeletal muscle and fat tissues. Elimination of p16^INK4a^ in these mice increased their longevity, delayed their typical degenerative phenotype and reduced the accumulation of senescent cells, supporting the role of p16^INK4a^ in senescence induction and age related pathologies [53]. Further progression was made in later experiments when rather than depleting p16^INK4a^ itself, p16^INK4a^-expressing cells were targeted using the INK-ATTAC transgene which enables elimination of p16^INK4^- positive cells upon administration of a drug. The experiment was done both in BubR1^H/H^ progeroid mice [54] and "naturally aged" one year old mice [55]. The inducible elimination of p16^INK4a^ expressing senescent cells increased mice longevity, and attenuated the progression of age related pathologies, such as lordokyphosis, cataract and adipose accumulation. These results add an important layer to our understanding of senescence. Not only prevention of senescence development, but also the elimination of the already senescent cells delays aging. Thus, pathologies associated with senescence are not related solely to the loss of function of the senescent cell itself, but rather to its gain of new functions and its effect on the surrounding tissues.

While organismal aging is virtually a universal phenomenon, there are some exceptions to this rule. Lobsters, for example, retain telomerase activity in all tissues, and their telomeres do not shorten. Indeed, they continue to grow throughout their life without becoming slower or weaker. They retain high proliferative capacity, and are able to regenerate whole limbs at advanced age [56]. In other words- they show almost no, or very slow, biological aging. It has been speculated that this results from a very slow rate of cellular senescence [57]. Studying senescence in lobsters and similar "immortal" species may shed some more light on its relations to aging.

### 3.2 The senescence- associated secretory phenotype

Albeit in essentially permanent cell cycle arrest, senescent cells remain metabolically active, and continue a significant crosstalk with their environment. Microarray analysis of senescent fibroblasts revealed an mRNA expression map that bears a strong resemblance to inflammatory wound healing gene expression pattern, which includes multiple cytokines, chemokynes, growth factors and proteases [58]. Later on, Campisi and others elaborated this observation and defined the senescence- associated secretory phenotype (SASP) [13] which connects the response of a single cell to damage to a more complex process of whole tissue remodeling. Indeed, over the past few years, SASP became one of the most important hallmarks of senescence, and a key factor in our understanding of its complicated and ambivalent relations with aging and cancer [59, 60].

**Figure 2.**
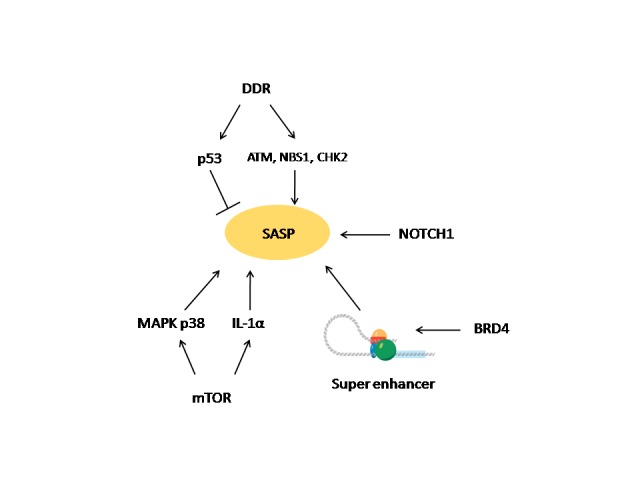
SASP regulation SASP is under a regulation of multifactorial singaling networks. The DDR effectors NBS1, ATM and CHK2 upregulate SASP. Importantly, p53 is a negative regulator of SASP and serves to restrain it upon DDR activation. mTOR positively regulates SASP via activation of the MAPK p38 pathway, and upregulation of IL-1α. Chromatin reorganization in senescent cells involves newly activated super enhancer (SE) elements. BRD4 is recruited to the SE and participate in the regulation of key SASP genes. NOTCH1 was indentified as a modulator of SASP composition in oncogene-induced senescent cells.

When comparing the molecular pattern of pre-senescent and senescent cells, SASP associated factors constitute a significant part of the changing pattern along with cell cycle and metabolism related genes. SASP includes several groups of factors that enable the senescent cell to modify its environment. These factors can be grossly divided into few major categories: soluble agents, secreted proteases and secreted extra-cellular matrix (ECM) components. The soluble factors include cytokines (IL-1β, IL-6), chemokines (IL-8) and various growth factors (HGF, TGFβ, GM-CSF) [13, 57, 61]. The SASP expression profile is plastic and varies between different tissues and different physiological contexts. However, some of the SASP elements, including IL-6 and IL-8, are highly conserved. Interestingly, both of these proinflammatory cytokines have been linked to aging-related pathology.

### 3.3. SASP regulation

SASP regulation is multifactorial (Fig. 2). It is induced mainly by DNA damage that triggers activation of the DDR, in parallel to the induction of cell cycle arrest. Specifically, it is upregulated by the DDR effectors NBS1, ATM and CHK2 [62]. It is only stimulated by persistent DDR and not by robust rapidly resolving damage. Importantly, p53 is a negative regulator of SASP, and so it serves to restrain SASP upon DDR activation [6].

Another important regulator of SASP is NF-κB [63], a key transactivator of multiple inflammatory genes, and a central mediator in inflammatory and tumorigenic processes [64].

Two recent studies pointed out the mammalian target of rapamycin (mTOR) as a master regulator of SASP. One study identified the MAPK p38 pathway as an mTOR dependent regulator of SASP [65]. Another study showed SASP regulation via inhibition of IL-1α [66]. IL-1α is a proinflammatory cytokine which is known as a regulator of SASP. Unlike its analog IL-1β which is a secreted cytokine that carries systemic inflammatory effects, IL-1α is membrane- bound and acts in an autocrine fashion [67]. IL-1α and NF-κB comprise a positive feedback loop that maintains SASP expression. The mTOR inhibitor rapamycin was found to inhibit the translation of IL-1α, and thus to down-regulate SASP [66]. As noted above, SA-β-Gal is a known marker of senescence, but its physiological significance remains poorly understood. Interestingly, *in vitro* treatment with rapamycin suppressed SASP along with SA-β-Gal, without affecting cell cycle. This may imply that SA-β-Gal is a surrogate of SASP rather than of the senescence associated cell cycle arrest [68].

Super enhancers (SE) are large regulatory elements in the chromatin that control multiple genes and contribute to synchronized downstream biological circuits [69]. The bromo-and extra terminal domain (BET) proteins are important transcription cofactors of genes that are under regulation of SEs. BET inhibitors are currently under development to suppress certain inflammatory and malignant diseases. BRD4 is BET protein that is important for myc expression in acute myeloid leukemia (AML) as well as the expression of other driver mutations in additional malignancies [70, 71]. A recent study identified reorganization of the chromatin in senescent cells with newly activated SE adjacent to key SASP genes. ChIP-seq analysis showed that BRD4 is recruited to the new SE and participate in regulation of critical SASP genes [72].

Notch signaling is involved in multiple developmental processes, cell- fate control, stem cells homeostasis, as well as stress responses and tumorigenesis [73]. Recently, NOTCH1 was identified as a regulator of the SASP composition in oncogene-induced senescence (OIS). NOTCH1 induced shifting of the senescent cell secretome from pro-inflammatory one to TGF-β dominant. Inhibiting NOTCH signaling lead to modulation of the immune surveillance and promoted the clearance of the OIS cells from the liver [74].

### 3.4. Pro- tumorigenic properties of a tumor suppressor mechanism?

In the early years of senescence study, the prevalent paradigm was that senescence is a strong tumor suppressing mechanism, which leads to permanent proliferation arrest in response to potentially oncogenic stressors. Consistent with this notion, senescent cells are found in pre-malignant tissues, both in mouse models and human benign tumors, yet almost absent in malignant ones [75]. In a mouse model of lung tumors, conditional activation of the oncogenic K-rasV12 allele results in the development of pulmonary adenomas, and some pulmonary adenocarcinomas. Staining for Ki67, p16 and SA-β-Gal showed a low proliferation index and high senescence signal in the pre-malignant adenomas, while the adenocarcinaoms showed high proliferation index with weak or no staining for senescence markers [76]. Additional example is the human nevi. They harbor the oncogenic allele BRAF^v600E^, but only rarely progress to malignant melanoma. This may be attributed to high senescence levels in these lesions [77].

Restoring the activity of tumor suppressors in malignant tissues was shown to induce a potent senescence response and tumor regression in some cases. E6AP is an E3 ligase which targets the tumor suppressor PML for proteasomal degradation. PML loss is a frequent event in prostate cancer. A study that examined knockdown of E6AP in both prostate cancer cell line and xenogrfts, showed that its down regulation resulted in PML accumulation, triggered efficient cellular senescence and lead to attenuation of tumor cells growth [78].

As the research on the field deepened, senescence was found to be a double-edged sword in its relations with tumorigenesis. While being, a strong tumor suppressing mechanism, accumulating evidence indicated that senescent is also a pro-tumerogenic factor in some cases [79, 80]. The understanding that senescent cells can alter their environment has raised the possibility that their accumulation in aged tissue might contribute to the exponential increase in cancer prevalence upon aging. Indeed, human senescent fibroblasts were shown to support malignancy. When injected to mice along with malignant epithelial cells, the senescent fibroblasts induced more aggressive tumors compared to epithelial cells that were injected with pre-senescent cells [81].

Research showed that SASP is the critical effector arm of the senescent cells regarding its environmental effect. SASP beneficial or harmful effect is context-dependant. On certain circumstances, it promotes wound healing, controls proper tissue remodeling and prevents progression of fibrosis as a response to damage [82-84]. In addition, SASP is important in recruiting immune cells which removed damaged and malignant cells in their surrounding tissue [85, 86]. However, on other circumstances SASP might promote malignancy.

As mentioned above, mTOR is one of the regulators of the SASP genes. *In vivo* study of prostate cancer xenografts found a better anti-tumoral effect of cytotoxic chemotherapy when the mTOR inhibitor rapamycin was added to the treatment [66]. One way to interpret these results is that rapamycin inhibited the chemotherapy-induced SASP, and so abrogated its pro-tumorigenic effect. In another study, senescence was induced *in vivo* in hepatocytes by expressing the oncogenic allele N-Ras^G12V^. Rapamycin treatment after the induction of senescence impaired the SASP, decreased immune cell recruitment and reduced the elimination of oncogenic hepatocytes. While in the former case SASP is assumed to have a pro-tumorigenic effect, in the later one SASP seem to have a tumor suppressor role as a mediator of efficient immune-surveillance [65].

In addition to SASP, another type of senescence- associated inflammatory phenotype was described. Senescence inflammatory response (SIR) is induced in senescent epithelial cells. Unlike SASP, SIR is mostly a cell autonomous response and contains only a small number of secreted factors. In a mouse model of colorectal cancer, it was shown that in the presence of intact WT p53 SIR serves as a tumor suppressor factor, but upon p53 loss SIR becomes a driver of tumorigenesis [87].

All the examples mentioned above emphasize the complexity of senescence and its related processes. Its anti- or pro-tumorigenic properties are highly dependent on the physiological context, and on various "biological switches" that determine if it goes one way or another; most of them are yet to be identified. While the cell cycle arrest that is associated with senescence is a crucial tumor suppressing mechanism, SASP is associated with many of the harmful pathologies. Understanding the different pathways that regulate SASP may enable us to target it specifically. The different cytokines, enzymes, and transcription factors mentioned above as regulators of SASP, including IL-1α, IL-6, mTOR and BRD4, are all potential targets for pharmacological inhibition, some of them already exist in the clinical practice, and may be exploit to mitigate the deleterious effects of SASP in the relevant clinical contexts.

## 4. The aging immune system and cancer

### 4.1. "Inflamm-aging" and cancer

Aging is associated with low- grade chronic sterile inflammation, so- called "inflammaging", which accompanies virtually all aging related disorders [14, 88, 89]. Epidemiologic data show correlation between elevated levels of inflammatory factors such as IL-6 and C-reactive protein (CRP) to multiple morbidities of the elderly. This inflammatory phenotype is not solely a biomarker, but rather a central biological theme in the aging process. It is thought to be the result of exposure to various endogenous and environmental insults throughout life, and in turn- a driving factor in multiple age-related pathologies [16, 90, 91].

Inflammation is a complex biological response to a harmful stimuli such as pathogen invasion, physical trauma or irradiation. Its function is to eliminate the harmful agents, repair the damaged tissue and restore homeostasis [92, 93]. While acute inflammation is an overt transient response to damage that is essentially beneficial and facilitates tissue repair, chronic inflammation is a low grade sustained process driven by continues stimulation, incomplete resolution of the stimuli or dysregulation of the immune response, and might ultimately results in tissue remodeling and dysfunction [94]. Chronic inflammation contributes to initiation and / or propagation of multiple pathologic processes, including degenerative disorders that accompany aging, and hyperplastic disorders including cancer [6, 95-97].

The various inflammatory responses that accompany aging involve both immune cells, especially macrophages [98], and non immune cells including fibroblasts and epithelial cells. There are several processes that stand in the basis of inflammaging. One source is the increased frequency of cellular senescence and its accompanying inflammatory components, IL-6 in particular. Another component in this chronic low grade inflammation is innate immune responses that results from various pro-inflammatory agents, and involve both immune and non-immune cells. These agents may be debris of macromolecules, different microbial components, DNA culprits or cytoplasmic DNA fragments, collectively known as danger-associated molecular patterns (DAMPs) [99]. Accumulation of DAMPs results in activation of innate immune receptors, which initiate immune responses that take part in the inflammaging network [100]. The first group of such innate sensors is the transmembrane pattern-recognition receptors of the Toll-like receptor (TLR) family [101]. TLRs signal via Myd88 and ultimately lead to activation of the pro-inflammatory transcription factors NF-κB and activator protein 1 (AP-1), upregulation of various inflammatory cytokines including TNF-α, IL-1β and IL-12, and activation of type I IFN immune response. Another group of receptors are the intracellular NOD-like receptors (NLRs). A prominent member of this group is the NLR pyrin domain containing 3 (NLRP3) that constitute a central part of the inflammasome complex. Inflammasomes are cytoplasmic protein complexes that use as a signaling hub for inflammatory responses. DAMP sensing by an NLR family member leads to assembly of the inflammasome complex and activation of caspase-1, which in turn lead to cleavage and secretion of mature proinflammarory cytokines, including IL-1β and IL-18 [102, 103]. A third group of innate receptors are the cytosolic DNA sensors that are activated by cytosolic dsDNA fragments and induce inflammasome signaling and type I IFN response [104]. Additional sources for inflammaging are increased activation of the coagulation system and inappropriate regulation of the complement pathway [88]. Another crucial component of the inflammatory responses in the aging process is the aging of the immune system itself, so called "immunosenescence", as will be further discussed.

Chronic inflammation contributes greatly to the development of age- related diseases, including cancer. One mechanism in which chronic inflammation promotes tumorigenesis involves the recruitment of myeloid-derived suppressor cells (MDSCs). MDSCs are heterogenic group of myeloid lineage-derived cells that posses immune-suppressive activity. In a melanoma mouse model, MDSCs were shown to accumulate in melanoma lesions and lymphoid organs. Their accumulation was associated with decreased expression of T cell receptor ζ chain and reduced anti-tumoral immune activity [105]. Breast cancer is an example for the strong connection between pro-inflammatory environment and malignancy. Specifically, it demonstrates the relation between IL-6, a major component of inflammaging, and cancer development and progression [106]. High serum levels of IL-6 in breast cancer patients correlates with poor prognosis [107]. Mamospheres (MS) are multicellular structures enriched with progenitor cells of the mammary gland. *Ex vivo* study showed an elevated IL-6 mRNA levels in MS obtained from aggressive ductal cell carcinoma, compared to MS from normal breast tissue [108]. Cancer stem cells represent subpopulation of tumor cells which are hypothesized to be key drivers of cancer. They are characterized by both self- renewal capacity, and the ability to differentiate into non-stem cancer cells. They are highly tumorigenic in xenograft experiments, and often resistant to standard chemotherapy [109, 110]. The IL-6/JAK2/Stat3 pathway was shown to be preferentially active in CD44^+^CD24^-^ breast cancer cells that harbor stem cell-like features [111]. In the MS model, IL-6 triggered Notch activation which contributed to cancer stem cell self renewal, promoted hypoxia survival and sustained aggressive invasive phenotype of the malignant cells [108]. In a different *in vitro* model, IL-6 stimulated non-stem cancer cells of breast and prostate cancer cell lines to gain cancer stem cells properties [112]. And so, breast cancer is one example for the contribution of IL- 6 rich environment, especially in the cancer stem cell niche, and it can serve to highlight the possible contribution of inflammaging to cancer propagation.

### 4.2. Immunosenescence

The classical hallmarks of cancer as were defined by Hanahan and Weinberg in 2000 were recently revised [113]. One of the new hallmarks that were added is the ability to escape from immune surveillance. This reflects the central part that immune responses to cancer carry today both in cancer research and in cancer treatment in clinical practice.

The immune system undergoes profound transformation with age which affects multiple aspects of immunity, including susceptibility to infections, autoimmunity, response to vaccination and cancer development [114, 115].

Aging is characterized by thymic involution, reduced T cell diversity, decrease in naive T cell population and increase in memory T cells [116, 117]. Loss of the co-stimulatory receptor CD28 is a hallmark of senescent T cells. This is at least partially attributed to chronic infection with cytomegalovirus (CMV) which results in constant antigenic stimulation and immune exhaustions [118]. NK cells are central component of the innate immune system. They too undergo phenotypic changes with age which may compromise their cytotoxic activity [119]. Along with macrophage age related changes and alternation in the γ/δ T cell population [120], age associated changes in T cells and NK cells affect greatly on the changing tumural microenvironment, and contribute to compromise immune surveillance.

The effect of the aging immune system on cell fate and tissue homeostasis was demonstrated in a mouse model of cutaneous squamous cell carcinoma (SCC). The conditional induction of mutant H-Ras in keratinocytes resulted in different outcome depending on the mouse age. While H-Ras activity was similar in young and old mice as was demonstrated by pERK staining, the outcome of its activation changed dramatically between the two populations. Young mice developed hyperplastic reaction and a rapid hair growth after hair shaving, whereas aged mice showed no acceleration of hair growth, developed dysplastic changes, and half of them developed SCC. As opposed to the young mice, the aged mice demonstrated a shift toward the pro-tomorigenic Th2 inflammatory response, increased expression of the immune checkpoint activator PD-L1, and increased SA-β-Gal staining in the dermis which probably represents senescent immune cells. These results demonstrate the marked changes in stem cell function, tissue regeneration capacity and fate of transformed cells in the context of young versus old immune system [121].

Recognizing the critical role of the immune system in tumorigenesis has brought immunotherapy to the frontline of anti cancer treatment. The idea of activating the immune system against cancer is several decades old. In the 1990’s IL-2 was approved by the FDA for treatment in renal cancer and melanoma, and became the first immunotherapeutic agent capable of achieving durable cancer response [122]. 15 years later, in 2006, a major set- back in the field has occurred. Healthy volunteers that were treated with anti-CD28 antibody in a phase I trial experienced a devastating cytokine storm response that necessitated their admission to an intensive care unit [123]. This was at least partly attributed to subtle but important differences between pre-clinical animal models to humans that failed the prediction of this reaction [124]. However, advances in immunology research allowed the recovery of immunotherapy, and it is now in the mainstream of modern oncology. Immunotherapy strategies include adoptive T cell transfer [125], chimeric antigen receptor (CAR) T-cell based therapy [126] and immune checkpoint inhibitors (ICI) [127]. The latter are currently the most common immunotherapeutic agents in clinical use, lead by CTLA-4 and PD-1 blockers. While cancer is mainly a disease of the old age, most of the pre-clinical data we have on immunotherapy relies on experiments done on young rodents. In a mouse model that examined the anti tumoral effect of combination therapy with andi-CD40 and IL-2, young mice achieved good response with metastatic tumor regression. However, old mice that got the same treatment suffered severe macrophage-mediated cytokine storm and died within 2 days [128]. These results highlight the critical importance of better characterizing both therapeutic and toxic effects of immunotherapy in the older population, In light of the profound changes in the immune system that accompany aging. A recent meta-analysis of randomized-controlled trials examines the efficacy of ICI among younger and older cancer patients [129]. When a cutoff point of 65-70 years was used, both younger and older patients showed similar improvement in overall survival and disease-free survival. However, in a subgroup of patients older than 75 years no beneficial effect of anti PD-1was seen. This further emphasizes possible effect of immunesenescence on anti-cancer treatment.

### 4.3. Autophagy in inflammaging and cancer

Autophagy is a highly evolutionary conserved mechanism for recycling of intracellular proteins and organelles. While it is an essentially self-degenerative process as its name- ’eating of self’ implies, it is now clear that autophagy is a pro-survival mechanism crucial for cellular adaptation to stress, as well as for quality control processes, regulation of immune responses and maintenance of tissue homeostasis [130, 131]. In the autophagy process, intracellular "waste products" such as misfolded proteins and defective organelles are recognized and sent to lysosomal degredation. In mammals, three types of autophagy are recognized: microautophagy, chaperone-mediated autophagy and macroautophagy. Microautophagy involves invagination of lysosomal membrane and direct engulfment of the target cargo. In chaparone-mediated autophagy, soluble cytosoloic proteins that contain a KFERQ-like pentapeptide motif are recognize by the chaperone protein HSC70 and sent to lysosomal degradation. Macroautophagy involves the generation of a double-membrane autophagosome that sequester cellular cargo that was tagged for autophagy. The autophagosome fuses with the lysosome to create the autophagolysosome in which the luminal content is degraded [132-135]. Macroautophagy, hereafter referred as ’autophagy’, is considered to be the dominant type in maintaining cellular homeostasis.

Due to its role in protein turnover and removal of cellular debris and dysfunctional organelles, autophagy serves as an anti-aging mechanism. Indeed, although the changes in autophagy with age remain largely elusive, accumulating evidence link a decrease in autophagy capacity with the aging process [136]. Atg genes encode a family of proteins that are essential for autophagy execution. In pre-clinical rodent models, whole body knockout of Atg genes resulted in early postnatal death due to the defect in mobilizing of intracellular energy reserves. Tissue-specific Atg knockout show multiple age associated stigmata, which indicate the importance of autophagy in age-related pathologies [134]. Multiple reports describe decline in autophagy related proteins in aged tissues. The important autophagy mediators ULK1, Beclin-1 and LC-3 were shown to be down-regulated in human chondrocytes taken from osteoarthritis patients [137]. Decrease in sirtuin-1, a longevity-associated protein and a positive regulator of autophagy, was linked to insulin resistance and the metabolic syndrome [138]. Decreased autophagy was also described in age-related cardiac pathologies [139]. In a work that examined transcription patterns of autophagy-related genes, significant changes were demonstrated in younger versus older human brain samples. In the aging brain, genes related to the MAPK pathway, which was linked to autophagy suppression, were up-regulated. Conversely, genes that are directly involved in the autophagy pathway, such as Atg5, Atg 7 and beclin-1 were down-regulated. Together these data suggest a decreased autophagy activity in the aging brain [140]. The autophagy pathway is a target for pharmacologic interventions that may modulate aging. The lifespan extending effect of caloric restriction is hypothesized to be mediated by increased autophagy. And so, caloric restriction mimetic compounds that stimulate autophagy are being developed as aging-modulating agents [141]. mTOR is a sensor of cellular nutritional status, and serves as an important coordinator that balance between cell growth and autophagy [142]. Longevity promoting by mTOR inhibitors is at least partially attributed to their autophagic enhancing properties [143]. Autophagy has a crucial role in removal of intracellular carcinogenic agents, maintenance of genome integrity and tumorigenesis prevention [144]. Thus, the decline in autophagy capacity with age seems to contribute to the process of tumor initiation. However, the role of autophagy in tumor progression bears great complexity. Being a pro-survival mechanism, autophagy may facilitate tumor cell survival. Cancer cells can up-regulate autophagy, which support their survival in the context of increased metabolic demands and hostile microenvironment [145]. On the other hand, recent studies have demonstrate that in addition to the cell autonomous properties of autophagy, it bears non cell-autonomous functions that modulating cancer-associated inflammation and immune-surveillance and result in anti-tumorigenic effect [132]. Autophagy serves to restrain DAMPs-induced inflammation by elimination of stimulating agents such as damaged mitochondria [146], and so mitigate its pro-tumorigenic effects. It also has an important role in anti-tumoral adaptive immunity. The autophagy machinery participates in antigenic processing and presentation by MHC class II molecules. It may also contribute to antigen presentation by MHC class I molecules, especially when the canonical pathway of pathogen proteasomal processing and peptide import to the ER by TAP is inhibited. In addition, autophagic exocytosis was suggested as a mechanism for antigen transfer and cross-presentation by antigen-presenting cells [147], which may play an important role in cancer antigen presentation. Over the past years, as research in cancer immunology deepened, it became apparent that the anti-cancer effect of classical treatments such as some conventional cytotoxic drugs and radiotherapy is partially immune-response mediated. This is postulated to be due to the induction of "immunogenic cell death" (ICD). Unlike apoptosis which is a silent form of cell death, ICD is an immunogenic process that stimulates immune response against antigens originating from the dying cell. It involves release of immunogenic factors such as HMGB1 and ATP that generate ’eat me’ signals and recruit immune cells [148]. ICD is now thought to be an important factor in efficient tumor immune-surveillance which may increase the therapeutic effects of anti-cancer treatments. DAMPs release by cancer cells in an autophagy-dependent manner is suggested to be an important factor in ICD. In line with this notion, it was shown that while cisplatin and oxaliplatin induce apoptosis in prostate cancer cell is similar level, only oxaliplatin, which stimulate autophagy to greater extent than cisplatin, triggers ICD [149]. Thus, autophagy recruitment was suggested as a therapeutic strategy to enhance cancer immunotherapy [132].

To conclude, autophagy decline with age contributes to multiple aging-related pathologies including cancer initiation. However, its role in tumor progression remains complex and contradictory.

## 5. Mincrobiome, aging and cancer

The bacterial population of the gut microbiome outnumbers human cell by approximately 10 fold [150], and is sometimes referred to as the ’forgotten organ’ due to its increasingly recognized role in multiple physiological and pathological processes[151]. Over the last decade, it was shown to have a pivotal function not only in the development of local intestinal pathologies such as inflammatory bowel disease, but also in systemic phenomena such as obesity, diabetes, cancer and various neurologic and psychiatric diseases [152-154]. The intestinal mucosa which includes epithelial cells, gut-associated lymphoid tissue (GALT) and overlying mucus layer, constitutes a mechanical, biochemical and immunological barrier between the microbiome and its host [155]. Barrier dysfunction results in altered interaction between commensal bacteria and the host, and thus contributes to the so-called ’sterile’ inflammation that accompanies many of the above-mentioned pathologies.

Aging related pathophysiological changes display a reciprocal interaction with the gut microbiome. Aging affects intestinal barrier function, and accompanied by alternation in the microbiome composition. These changes, in turn, modulate various age related disorders. The correlation between microbiome, barrier function and aging was demonstrated in several experimental models. In a Drosophila model, intestinal barrier integrity was shown to be reduced with age and upon exposure to increased oxidative stress. Barrier dysfunction in the flies was linked to metabolic dysfunction, increased expression of inflammatory genes and reduced physical activity [156]. Caenorhabditis elegans (*C. elegans*) is a powerful model for studying many aging related pathways. Several studies showed changes in C elegans longevity depending on the type of co-cultured bacteria and the presence of specific bacterial products [157]. Evidence that link aging and dysbiosis exist in human as well. An important player in this aspect is the aged gastrointestinal (GI) tract. Aging-related GI changes include decreased intestinal motility, high prevalence of diverticular disease, changes is salivary function and poor dentition. These intrinsic functional changes join environmental factors such as exposure to multiple medications that may alter GI function, and nutritional changes associated with old age, all of which contribute to changes in the GI lumen conditions and may lead to dysbiosis [158]. Congestive heart failure (CHF) is one of the most debilitating diseases of aging. Disturbance in the intestinal microcirculation and nonocclusive mesenteric ischemia that is associated with CHF result in hypoperfused edematous intestinal mucosa. This impairs mucosal integrity, and alters the local mucosal pH and redox state. These conditions might increase adherent commensal bacteria invasiveness and induce local proinflammatory cytokines. It also allows penetration of bacterial lipopolysaccharide (LPS) which triggers systemic secretion of various cytokines including TNFα, IL-1 and IL-6, which act as cardiosupressors and further exacerbate CHF. They also contribute to inflammaging and its associated pathologies [159].

A study that compared the microbiota of adults aged 65 and older to that of younger adults show that Bacteroidetes was the dominant phylum in the majority of individuals over 65, as opposed to the younger group where Firmicutes was the dominant phylum in most cases [160]. In a different study focusing on 178 elderly subjects aged 64-102, the association between microbiome composition and various lifestyle variants was examined. Bacteroidetes dominance was shown to be more prevalaent among frail long term residential care individuals, who also had higher inflammatory markers, including TNF, IL-6, IL-8 and CRP, and tend to consume high fat low fiber diet. Healthier community dwellers who had lower inflammatory markers and consumed low fat high fiber diet, had more diverse microbiome population with Fimicutes being the dominant phylum [161].

While the association between cancer and bacteria was described decades ago, the causal relations between the two have been debatable. However, accumulating evidence suggest bacteria as a ’driver’ force in tumorigenesis rather than solely a passive ’passenger’ factor [15]. An example for such association is Fusobacterium nucleatum which is significantly more prevalent in human colorectal adenomas and adenocarcinomas compared to the normal adjacent tissue. It promotes tumor development by creating a protumorigenic inflammatory environment via recruiting myeloid cells which promote tumor progression [162], and by inhibiting anti-tumor cytotoxic activity of NK cells [163].

The exact association between specific microbial composition, cancer and aging is yet to be defined, but there is some correlative evidence that support the notion that the microbiome is an important player in aging related cancer. Bacteroidetes phylum which is dominant among aged frail individuals tends to be more prevalent among colorectal cancer patients compared to health controls [164]. The inflammatory responses triggered by aging associated dysbiosis and intestinal barrier dysfunction seem to be an important contributor to inflammaging and thus contribute to a protumorigenic environment in the host which exceeds beyond its local effect in the intestine.

## 6. MicroRNAs, cancer and aging

MicroRNAs (miRs) are small non- coding RNAs that play a key role in the post-transcriptional regulation of many genes. It is estimated that more than 50% of human protein coding genes are regulated by miRs. Having a relatively low binding specificity, a single miR can target dozens of different genes, which place them as master regulators of multiple signaling pathways [165-167]. MiRs activity goes beyond the specific cell in which they are expressed. They can be transferred to adjacent cells via gap junction. They also reach distant cells in a non-contact dependant manner via microvesicles that are released to the microenvironment or to the blood stream. These properties make them possible candidates for being important regulators of complex systemic processes such as aging, systemic inflammatory responses, tumorigenesis and metastatatic spread. Indeed, several miRs were connected to cellular senescence, age related inflammation and cancer [168, 169].

MiR-21 is an example for an onco-miR which is over expressed in multiple human tumors [170]. It downregulates the tumor suppressor PTEN, induces tumor angiogenesis via enhancing VEGF expression, and seems to have a role in DNA damage-induced NF-κB activation [171-173]. In addition to its role in tumorigenesis, miR-21 has pro- inflammatory properties including promoting monocyte adhesion, and suppression of anti-inflammatory cytokines such as IL-10 and TGF-β [174]. A study that examined circulating miR levels in healthy volunteers aged 20-105 found statistically significant higher levels of miR-21 in octogenarians compared to the young group. Further analysis showed higher miR-21 levels in patients with cardiovascular disease compared to aged-matched healthy controls [175]. This highlights miRs as promising biomarkers, as well as potential novel therapeutic targets for cancer treatment and other age-related morbidities [176].

## 7. Cancer and aging- possible lessons from centenarians

Whereas cancer prevalence increases exponentially after the age of 65, demographic studies show it reaches a plateau at around 85, and then starts to decline [177, 178]. This deviation from the trend line may imply a unique biological behavior in this group of the very old, which help them to evade from carcinogenesis.

While cancer related death rate decreases dramatically after the 9^th^ decade to 0-4% after the age of 100, some autopsy studies actually demonstrate a continuous elevation of cancer prevalence with age, and increased prevalence of multiple primary tumors. However, cancer in the older ages tends to be less metastatic, and more often discovered as an incidental finding of a latent tumor. This imply that the lower prevalence of cancer that arises from epidemiologic studies actually represents lower rate of clinically significant disease and a less aggressive tumor behavior, and not necessarily decrease in tumor initiation [179].

Genetic polymorphism studies that examined changes in various tumor suppressor genes failed to identify a clear correlation between specific polymorphisms frequencies and cancer protection in centenarian. P53 polymorphism does seem to modulate cancer risk in context of high levels of environmental stress, but it does not clearly expressed in elevation of the relevant allele frequencies in centenarians [178]. It was suggested that changes in innate immunity and expansion of certain sub-population of lymphocytes expression NK receptors with potent anti-tumoral activity creates hostile environment for neoplastic growth in the oldest-old [180]. Few studies that examined miR expression profile found a significant overlap between young individuals and centenarians, which differ from the expression among octogenarians. This may suggest gene expression pattern that provides protection against age related morbidities including cancer [181]. The decline in autophagy capacity with age may be another pre-cancerous deficiency which protects centenarians against tumorigenesis.

The mechanism behind the relative resistance of centenarians to cancer is still poorly understood, but further study of this unique population may sharpen our understanding of the mechanisms behind aging related cancer.

## 8. Conclusions

The dramatic increase in the average life expectancy over the last decades has brought aging related pathologies to the center of biological and medical research interest. Along with cardiovascular diseases, cancer is the leading cause of death in the western world, and its importance as a cause for morbidity and mortality is predicted to grow even further as the population continues to age.

Extensive research in the area of aging and cancer in the past two decades has yielded a great advance in our understanding of the field. One of the most important milestones was the discovery of the dichotomic relations between cellular senescence and cancer. While being an essential tumor-suppressing pro-survival mechanism, prolonged presence of senescent cells promote tissue degeneration and may contribute to tumorigenesis via its associate inflammatory responses such as SASP or SIR. The accumulation of senescent cells in aged tissues is thus suggested to be a key factor underling age related cancer. Senescence-associated inflammation joins to the low grade inflammatory responses triggered by various DAMPs, to autophagy-related immune changes and to the changing profile of the aging immune system, to create an inflammatory network that accompanies aging and age-related pathologies. This inflammatory environment is hypothesized to create pro-tumorigenic conditions that make aged organisms to become more vulnerable to oncogenic insults.

Understanding the molecular mechanisms behind these processes may help to direct future anti cancer treatment. Long term aspirin and NSAIDs use were suggested to decrease cancer incidence due to their anti-inflammatory effect [57, 182, 183]. BRD4, mTOR, IL-1α and additional regulators of SASP are potential targets for anti cancer treatment. Chemotherapy-associated senescence can contribute to treatment success due to induction of cell cycle arrest, but it might also cause tumor resistance, or even paradoxical tumor progression under treatment mediated by SASP. Adding an adjuvant ’SASP inhibiting’ treatment to the conventional therapy may increase its efficacy. Various inflammatory mediators including miRs and cytokines such as IL-6 and IL-8 are too potential therapeutic targets. Finally, immunotherapy that will improve senescent cell clearance is another possible way to modulate age related cancer development.
